# Antibiotic tolerance and persistence in clinical isolates of *Escherichia coli* evaluated by high-resolution time-kill assays

**DOI:** 10.1128/spectrum.01124-25

**Published:** 2025-08-07

**Authors:** N. R. Alexandersen, K. L. Nielsen, S. Häussler, T. Bjarnsholt, K. Schønning

**Affiliations:** 1 Department of Clinical Microbiology, Copenhagen Universisty Hospital, Rigshospitalet, Copenhagen, Denmark; 2 Faculty of Health and Medical Sciences, Costerton Biofilm Center, University of Copenhagen, Copenhagen, Denmark; 3 Twincore, Center for Experimental and Clinical Infection Research, a Joint Venture of the Helmholtz Centre for Infection Research and the Hannover Medical School, Braunschweig, Germany; 4 Department of Clinical Medicine, Faculty of Health and Medical Sciences, University of Copenhagen, Copenhagen, Denmark; Innovations Therapeutiques et Resistances, Toulouse, France

**Keywords:** time-kill curves, persistence, tolerance, *Escherichia coli*, beta-lactams, quinolones, mathematical modeling

## Abstract

Bacteria showing tolerance to antibiotic killing are characterized by a slower kill rate compared with other isolates from the same species. Antibiotic persistence is defined by a subpopulation of bacteria showing a reduced kill rate when an isolate is exposed to a bactericidal antibiotic. Both are best studied using time-kill experiments and have been associated with treatment failure and the development of antibiotic resistance. Despite their potential clinical importance, quantitative data for the variation in antibiotic tolerance and persistence are lacking. Here, we performed time-kill experiments using 15 clinical *E. coli* isolates with or without acquired TEM-1 or OXA-1 β-lactamases and *E. coli* ATCC25922 against four antibiotics (piperacillin-tazobactam, cefotaxime, meropenem, and ciprofloxacin) at 10× minimum inhibitory concentration (MIC) in a rich medium. We used mathematical modeling to obtain quantitative estimates of tolerance and persister frequency. For β-lactams, the time-kill curves showed three distinct phases: an initial bacteriostatic phase, a second phase with rapid killing, and finally, a phase with slow killing characteristic of the persister fraction. For ciprofloxacin, the initial bacteriostatic phase was absent. Estimates of the duration of the bacteriostatic phase and fast kill rates were correlated between piperacillin-tazobactam and cefotaxime, but not with meropenem or β-lactamase content. Using MDK_99_, that is, the duration of antibiotic exposure to obtain a 2 log reduction in CFU count, as a measure of tolerance, we did not identify highly tolerant isolates. MDK_99_ was the shortest for ciprofloxacin. The proportion of cells undergoing slow second-phase killing correlated between all β-lactams and was highest for meropenem. Only 1/15 clinical isolates displayed an increased proportion of persister cells.

**IMPORTANCE:**

Studies of the clinical impact of antibiotic tolerance and persistence have lacked standardized protocols and definitions. This study uses reference methodology and mathematical modeling to provide quantitative measures in clinical *E. coli* isolates of metrics commonly used to describe tolerance and persister cell frequency. We observe that the β-lactam-mediated killing is preceded by a bacteriostatic phase that was absent for ciprofloxacin. The data indicate that tolerance may be specific for the mechanism of antibiotic action, and persister cell frequency varies for different drug classes. Using the data obtained from the study isolates, we do not observe increased tolerance for any isolate for the antibiotics tested, and only a single isolate displayed a high persistence phenotype. This study provides a basis for obtaining quantitative definitions of isolates showing high degrees of tolerance and/or persistence.

## INTRODUCTION

Antibiotic tolerance is defined as the increased survival of a bacterial population compared with a wild-type population when challenged with high concentrations of bactericidal antibiotics (1, 2). Tolerance differs from resistance in the inability of tolerant bacteria to replicate at these high concentrations of antibiotics. In MIC-based susceptibility testing, tolerant bacteria do not differ from wild type.

Tolerance is a population-wide phenomenon conferring increased survival to the main bacterial population when exposed to a bactericidal antibiotic. If only a small subpopulation displays drastically increased transient survival compared with the main population, this is defined as antibiotic persistence, and the cells belonging to this subpopulation are referred to as persister cells (2, 3). Bacterial strains can be labeled as having either high persistence or low persistence, depending on the size of the subpopulation compared with the main population. Persister cells are genetically identical to the main population, and the increased survival of the subpopulation in the presence of an antibiotic may be attributed to the persister cells being in a state of dormancy (4, 5). Persister cells are able to reactivate and reproduce after the termination of antibiotic exposure, allowing the surviving bacteria to reestablish the initial population (5).

A general reduction in bacterial growth rate is associated with a concomitant reduction in antibiotic kill rate, which may be interpreted as tolerance (1, 6). Uniform and standardized growth conditions are therefore necessary to study whether different bacterial strains differ in tolerance or persistence.

Both tolerance and persistence are suspected causes of antibiotic treatment failure and recurrent infection because of the increased survival caused by tolerance and persistence in themselves (1, 2, 4, 5). Furthermore, the development of tolerance may precede antibiotic resistance in *in vitro* evolution studies (7–9). Unfortunately, neither phenomenon can be detected in the diagnostic routine in a clinical microbiological laboratory (2). The current gold standard for measuring both tolerance and persistence is time-kill assays, which are too labor- and time-consuming to be practical in a clinical laboratory. A suggested alternative is the more convenient TDtest (10, 11), which does not discern between tolerance and persistence or provide quantitative measurements (1, 12). Many, especially older, studies used the MBC/MIC ratio to determine tolerance, which is generally considered to be inferior to and has a poor correlation to time-kill assays (1). In addition, even comparing studies using time-kill assays can be a challenge because of the lack of consensus on how to conduct the assay or interpret its results. Tolerance manifests in time-kill assays either as periods without any bacterial killing or as a reduction in the kill rate during kill phases (2). In a consensus statement, it was proposed to quantify tolerance using the minimum duration of killing 99% (MDK_99_), which is the time required to obtain a 2 log bacterial reduction (99% reduction) (2). Persistence in a time-kill assay is characterized by the presence of at least two distinct kill phases (2, 12, 13). The first kill phase represents the killing of the main population and displays a fast kill rate, whereas the second kill phase represents the slow killing of the minority population of persister cells (2, 3, 12). The poor comparability between studies, in addition to a low number of studies providing quantitative measurements, means there is no consensus for quantitative definitions of tolerance and persistence.

In this study, we aim to provide quantitative measurements of tolerance and persistence to define the distribution of these metrics in clinical isolates of *Escherichia coli* challenged with meropenem (MEM), cefotaxime (CTX), piperacillin/tazobactam (TZP), and ciprofloxacin (CIP). Having previously observed that overproduction of β-lactamase may cause resistance to penicillin/β-lactamase inhibitor combinations (14), we speculated that TZP tolerance would be more frequent among β-lactamase-producing isolates. We therefore included isolates with and those without acquired β-lactamases.

We determine tolerance and persistence using a high-resolution time-kill assay with frequent sampling, especially during the first 1.5 h of the experiment. We aim to establish a reproducible method, which allows us to establish distributions of values for tolerance and persistence among wild-type isolates to enable a separation of tolerant or persistent strains from wild-type strains. We find that isolates showing antibiotic tolerance or high-level persistence are infrequently occurring when exposed to β-lactams or ciprofloxacin at high multiplies of MIC and do not depend on the production of acquired β-lactamases, but that ciprofloxacin and β-lactams preferentially binding penicillin binding protein 2 (PBP2) or penicillin binding protein 3 (PBP3) all differ in their bacterial kill pattern.

## RESULTS

### Time-kill assay

Time-kill assays were done for 15 clinical *E. coli* strains and *E. coli* ATCC25922 using log phase cells exposed to an antibiotic concentration of 10× MIC as determined in a broth macrodilution assay using the same inoculum as in the time-kill assay. Frequent sampling every 15 min was done for the first 90 min. Untreated control cultures showed exponential growth during the first 75 min of the assay with doubling times between 25 and 30 min (Fig. S1), confirming the log phase of the inoculum.

**Fig 1:**
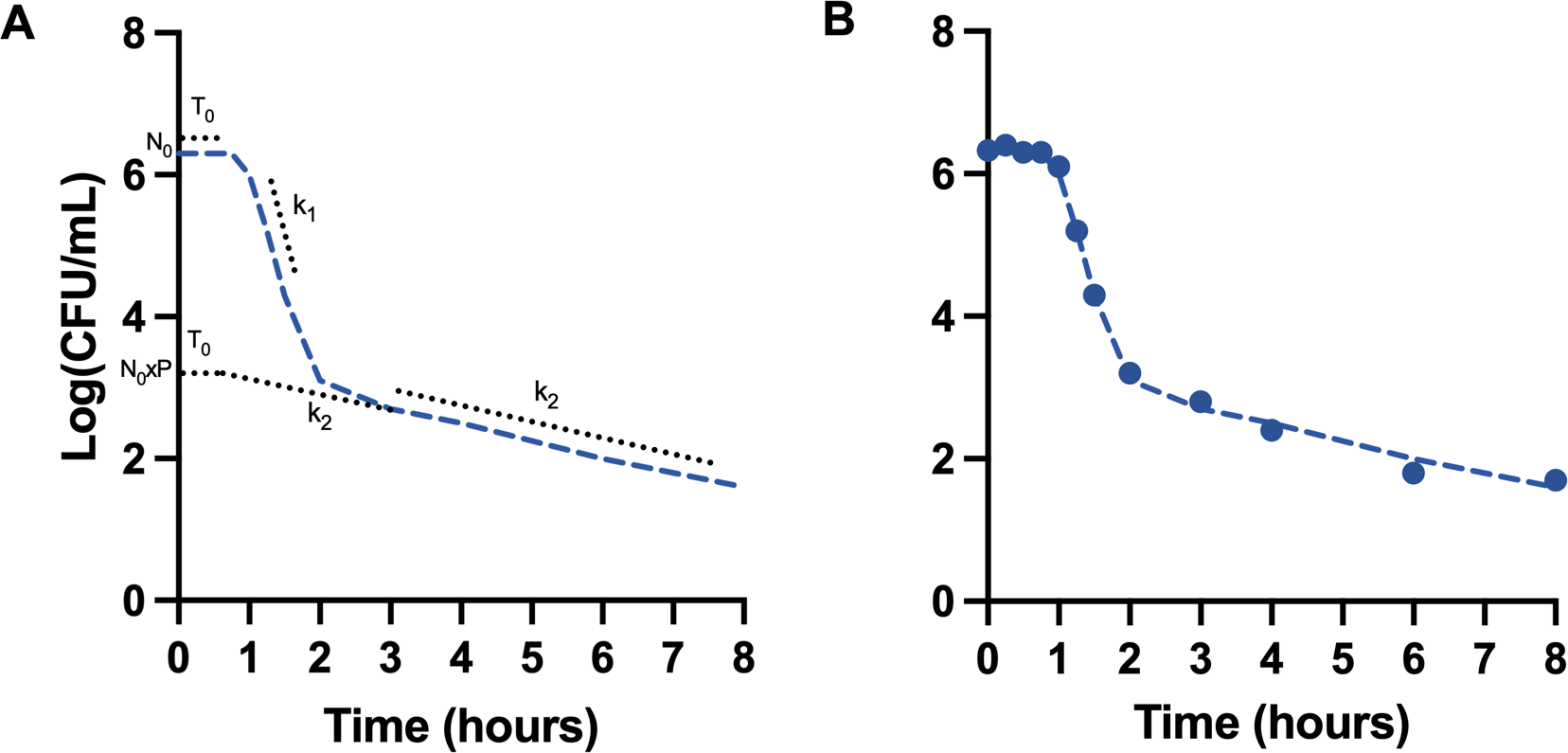
Experimental kill curve data identified three distinct phases: (i) a bacteriostatic phase (duration *T*_0_), and (ii) a rapid kill phase characterized by the kill rate (*K*_1_) of the main population, and (iii) a slow kill phase characterized by the kill rate (*K*_2_) of the persister subpopulation. The left panel (**A**) depicts a theoretical kill curve, and the right panel (**B**) shows the experimental data of an experiment using *E. coli* ATCC25922 and cefotaxime at 10× MIC. In addition to *t*_0_, *k*_1_, and *k*_2_, the size of the inoculum (N_0_) and the fraction of cells undergoing slow kill (*P*) were also fitted to experimental data in the mathematical model.

The results of a typical time-kill assay with CTX at 10× MIC on *E. coli* ATCC25922 are shown in Fig. 1. For time-kill assays using β-lactams, three distinct phases were apparent: First, a bacteriostatic phase without any reduction of CFU counts; second, a phase of rapid reduction in CFU counts; and finally, a phase with a slow reduction in CFU counts. The results were modeled mathematically as two populations undergoing exponentially killing with different kill rates following an initial bacteriostatic phase allowing optimization of the following parameters: *N*_0_ (inoculum size), *T*_0_ (duration of the bacteriostatic phase), *k*_1_ (kill rate of the population undergoing rapid killing), *k*_2_ (kill rate of the population undergoing slow killing), and *p* (the proportion of inoculum cells undergoing rapid killing) (Fig. 1). The proportion of cells undergoing slow, second-phase killing was used to estimate persistence. Two estimates for tolerance were used: (i) the half-life of cells undergoing rapid, first-phase killing, and (ii) the minimal duration of kill reducing viable cells by 2 log (MDK_99_).

### β-lactam-mediated killing of *E. coli*

**Fig 2:**
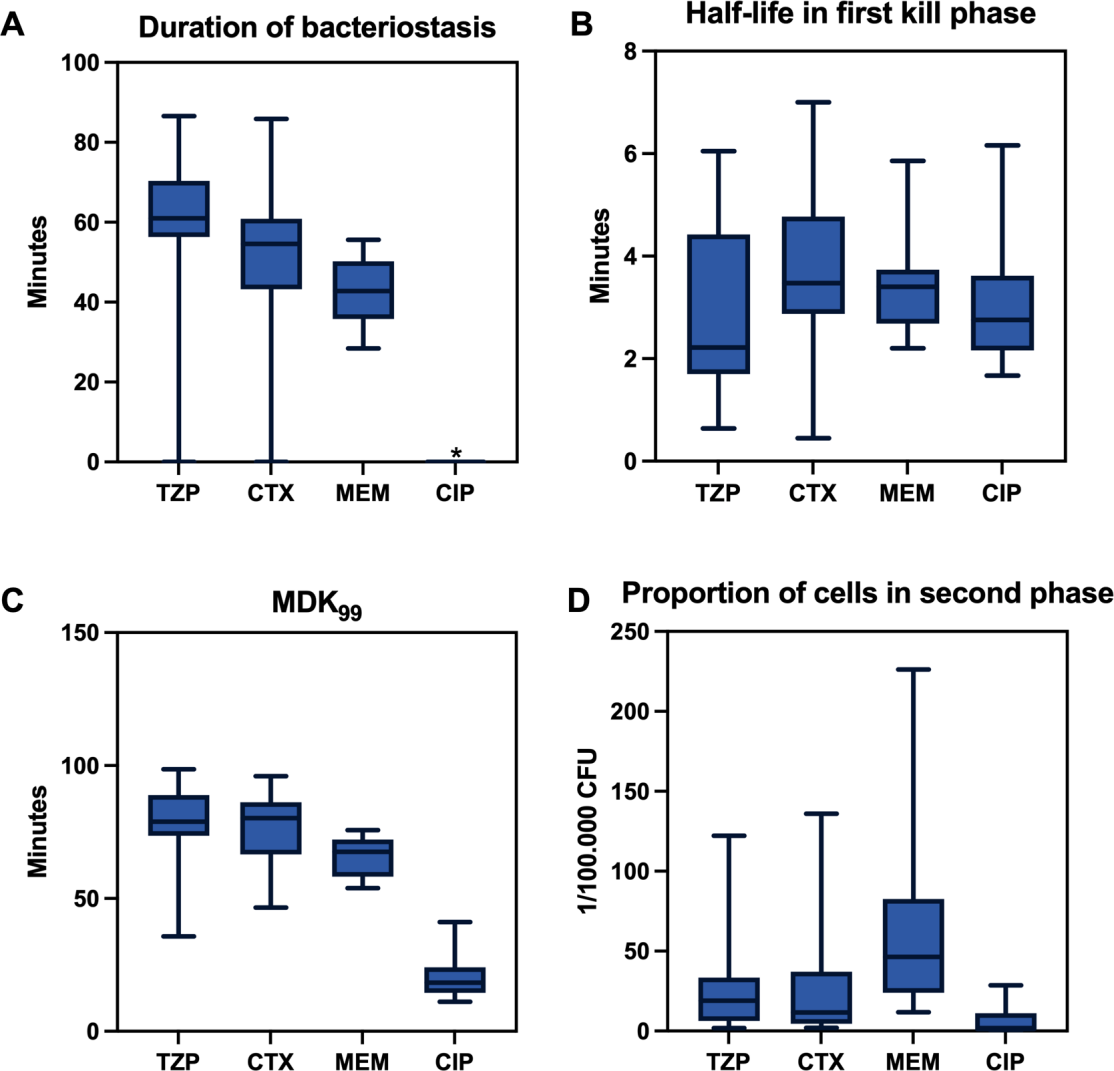
Box plots depicting the variation in kill metrics of clinical isolates of *E. coli*. The duration of the initial bacteriostatic phase (**A**), the half-life of CFU counts in the first kill phase (**B**), the MDK_99_ (**C**), and the proportions of cells undergoing slow kill (**D**) are shown as box and whiskers plots. Boxes indicate 25% and 75% percentiles. Whiskers indicate minimum and maximum values. The asterisk (*) in the upper right panel marks that for CIP experimental data, the data were fitted without an initial bacteriostatic phase.

**Fig 3:**
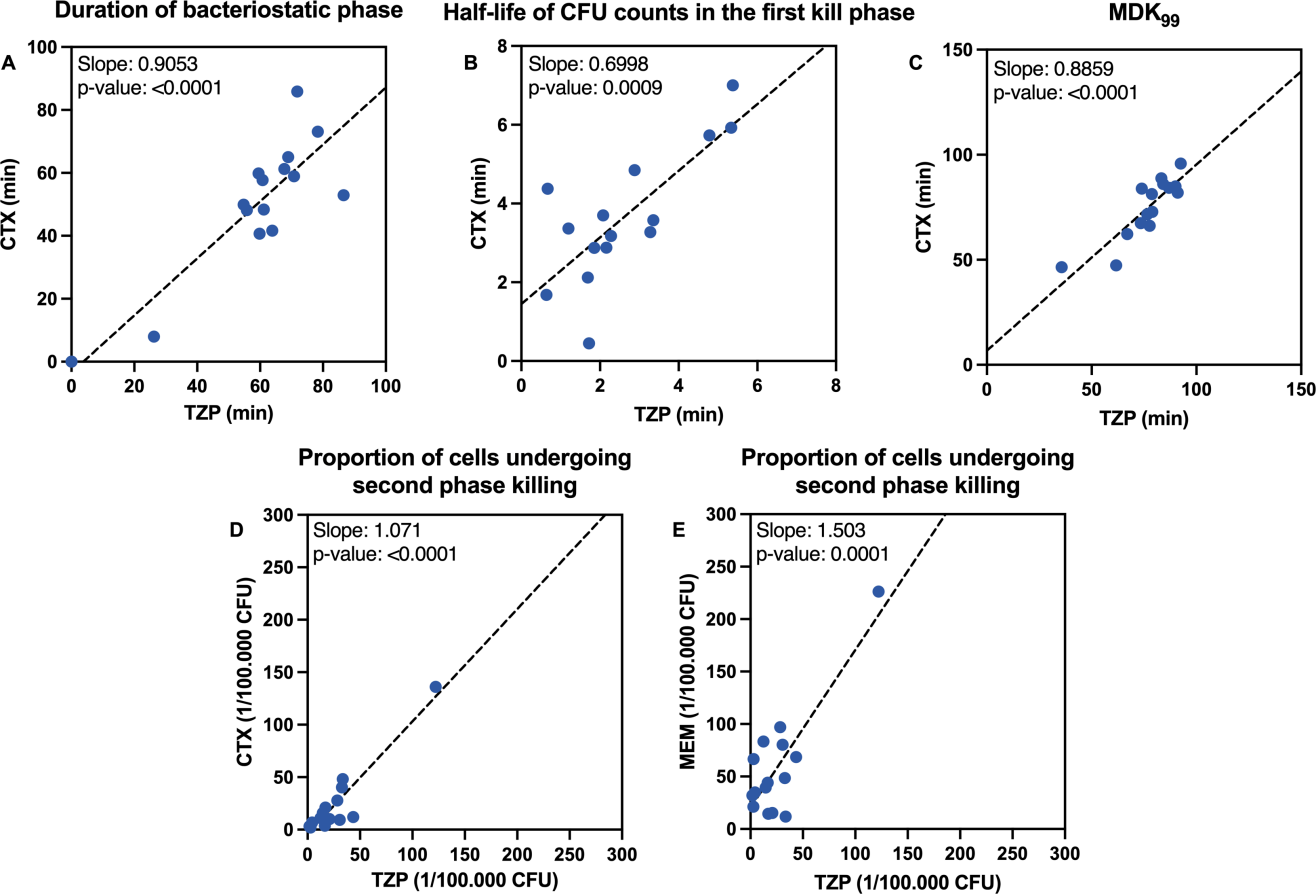
Correlations of kill metrics. The duration of the bacteriostatic phase (**A**), the half-life of cells undergoing rapid killing (**B**), and the MDK_99_ were correlated between TZP and CTX (**C**). The proportion of cells undergoing the second phase, slow killing, was correlated for all β-lactams. Shown are the correlations between TZP and CTX (**D**) and TZP and MEM (**E**).

Time-kill experiments using 10× MIC of TZP, CTX, and MEM were done in three biological replicates against 15 clinical isolates of *E. coli* and *E. coli* ATCC25922. Distribution of estimated kill curve parameters are shown in Fig. 2 and average parameter estimates after exclusion of outliers are summarized in Table 1. All strains displayed an initial bacteriostatic phase when exposed to MEM, and all but one strain had a bacteriostatic phase after exposure to CTX and TZP (Fig. 2A). After exclusion of outlier values, the mean duration of the bacteriostatic phase was 66 min (95% CI: 61 min to 72 min) for TZP, 57 min (95% CI: 50 min to 65 min) for CTX, and 43 min (95% CI: 38 min to 48 min) for MEM. The duration was significantly shorter for MEM than for TZP (*P* = 0.040) and CTX (*P* < 0.001) (Friedman test followed by post hoc Wilcoxon signed-rank test with Bonferroni correction). The duration of the bacteriostatic phase obtained for TZP correlated significantly with the duration of CTX (*P* < 1 × 10^−4^) (Fig. 3). This correlation was mainly caused by two outlier isolates with very short or absent bacteriostatic phases, as the correlation lost significance if the two isolates were omitted from the analysis. There was no significant correlation between TZP and MEM (Fig. S2).

**TABLE 1:** Average kill metrics on exposure to 10× MIC of piperacillin/tazobactam, cefotaxime, and meropenem^*a*^

	Duration of bacteriostasis	Half-life in first kill phase	MDK_99_	Proportion of cells in second kill phase
	(T_0_ – min)	(ln2/k_1_ – min)	(min)	(*P* – n/10^5^ CFU)
TZP	66.2 (60.6–71.7)	2.6 (1.7–3.5)	79.7 (74.5–84.9)	18.7 (11.0–26.5)
CTX	57.2 (49.6–64.8)	3.7 (2.7–4.7)	76.8 (69.4–84.2)	15.4 (7.2–23.6)
MEM	43.4 (38.4–48.4)	3.2 (2.9–3.5)	65.3 (62.4–70.3)	46.7 (30.8–63.1)
*P*-value	2.0 × 10^−4^	2.5 × 10^−3^	8.3 × 10^−3^	0.013

^*a*^ Mean values from 15 clinical isolates after exclusion of outlying values; 95% CI is indicated within parentheses. *P*-values were calculated using the Friedman test.

**Fig 4:**
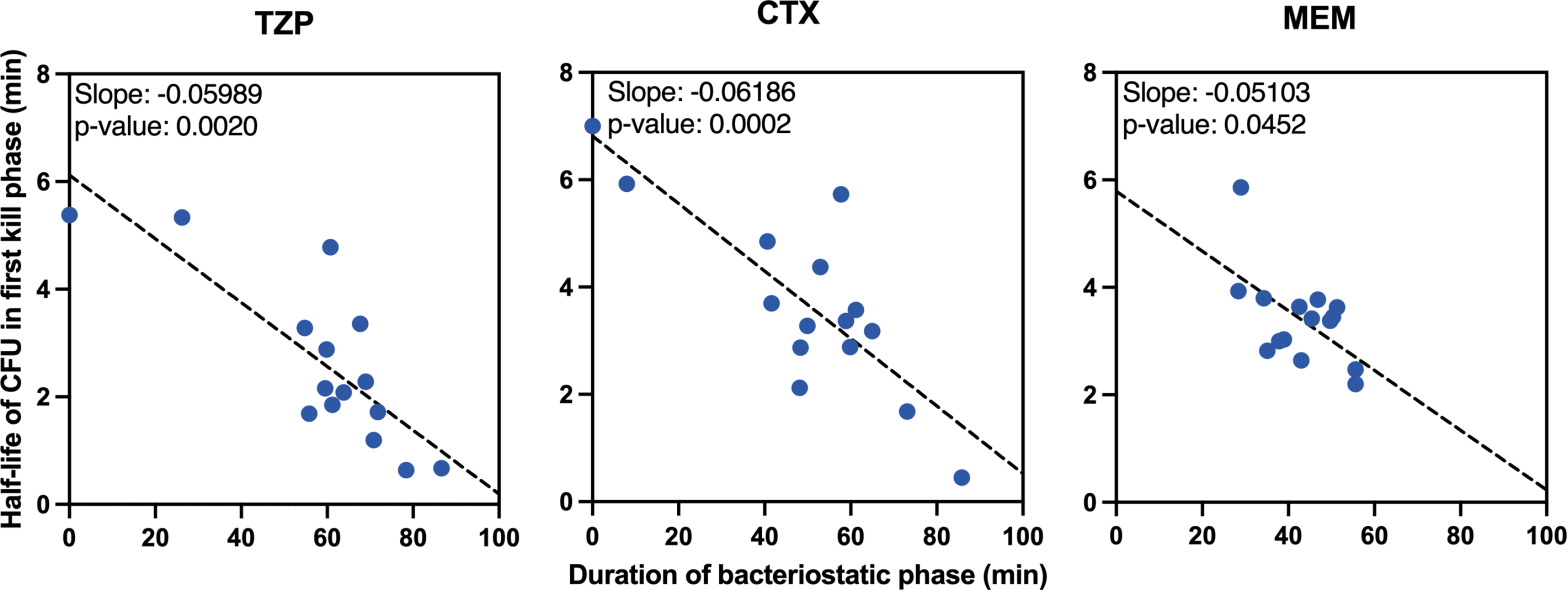
The half-life of cells during the first rapid kill phase was inversely correlated to the duration of the bacteriostatic phase, that is, a long bacteriostatic phase was correlated to a following rapid kill phase. Depicted are the correlations for TZP (left panel), CTX (middle panel), and MEM (right panel).

The average half-life of CFU counts during the first rapid kill phase was 2.6 min for TZP (95% CI: 1.7–3.5 min), 3.7 min (95% CI: 2.7–4.6 min) for CTX, and 3.4 min for MEM (95% CI: 2.9–3.9 min) (Fig. 2). The half-life was significantly shorter for TZP than for CTX when excluding outliers (*P* = 0.02; Wilcoxon signed rank test). The half-lives for TZP correlated significantly with CTX (*P* = 1.6 × 10^−3^) (Fig. 3), but not with MEM (Fig. S2). The half-life displayed a significant inverse correlation with the duration of the bacteriostatic phase for all three beta-lactams (*P* = 2 × 10^−3^ for TZP, *P* = 2 × 10^−4^ for CTX, and *P* = 0.045 for MEM) (Fig. 4).

Average MDK_99_ was not significantly different between TZP (80 min; 95% CI: 70–85 min) and CTX (77 min; 95% CI: 69–84 min) (Fig. 2), when excluding outliers. This was at least partly explained by a correlation between the duration of the bacteriostatic phase and the kill rate during the rapid kill phase. In contrast, for MEM, MDK_99_ (65 min; 95% CI: 62-70 min) was significantly shorter than for TZP MDK_99_ (*P* = 0.01, Wilcoxon signed-rank test) when excluding outliers, reflecting the shorter durations of the bacteriostatic phase obtained for MEM. The MDK_99_ of TZP and CTX correlated significantly (*P* < 1×10^−4^) (Fig. 3), but not to MEM (Fig. S2).

The average proportion of cells undergoing slow second-phase killing did not differ between TZP (19/100,000 CFU; 95% CI: 11/100,000 CFU – 26/100,000 CFU) and CTX (15/100,000 CFU; 95% CI: 7/100,000 CFU – 24/100,000 CFU) (*P* = 1.0; Wilcoxon signed rank test). For MEM, the average proportion of cells undergoing second-phase killing was 47/100,000 CFU (95% CI: 31/100,000 CFU – 63/100,000 CFU), which was significantly higher than for TZP (*P* = 0.012; Wilcoxon signed rank test). The proportion of persister cells obtained with TZP correlated significantly with both CTX (*P* < 1 × 10^−4^) and MEM (*P* < 1 × 10^−4^) (Fig. 3), although the correlation with MEM did not reach statistical significance if the outlier high persister isolate was excluded.

For 11 of 15 of the clinical isolates, kill metrics fell within 2 SD of the mean values provided above in all parameters during beta-lactam exposure. Of the remaining four isolates, one isolate did not display a bacteriostatic phase during treatment with TZP and CTX but showed a bacteriostatic phase within 2 SD of the mean when exposed to MEM (28 min). This lack of a bacteriostatic phase was reflected in the MDK_99_ for TZP (36 min), which was more than 2 SDs below the mean. In contrast, for this isolate, the MDK_99_ for CTX and MEM was both within 2 SD of the means (CTX: 47 min. and MEM: 55 min). A second isolate displayed a short duration of the bacteriostatic phase for TZP and CTX (26 and 8 min, respectively) but retained a typical bacteriostatic phase for MEM (51 min). A third isolate showed an increased half-life in the first kill phase for MEM (5.9 min). The last isolates displayed an increased persistence phenotype with 122/100,000 CFU, 136/100,000 CFU, and 226/100,000 CFU undergoing slow second phase killing for TZP, CTX, and MEM, respectively. Substitutions in *hip*A, G22S, D291A, and P86L confer a high persistence phenotype and are frequently identified in clinical *E. coli* isolates (15). We did not identify these substitutions in the isolates included in our study.

None of the clinical isolates displayed increased tolerance, defined as a significant increase in the MDK_99_.

Variation in tolerance and persistence was observed among clinical isolates of *E. coli* selected for this study. These had been chosen to represent isolates with acquired TEM-1 and OXA-1 β-lactamases and without acquired β-lactamases. When stratifying estimates of the duration of the bacteriostatic phase, half-life in the first kill phase, MDK_99_, and the proportion of cells undergoing second phase killing, we observed no significant difference between these three groups, with the exception of the proportion of cells in the second kill phase with CTX (*P* = 0.011; Kruskal-Wallis test). The results obtained for CTX are shown in Table 2. The results for TZP and MEM are available in Supplementary Data Tables S1 and S2. This indicates that killing β-lactam/β-lactamase inhibitor combinations is largely unaffected by TEM-1 and OXA-1 β-lactamases if β-lactam/β-lactamase inhibitor is present at high multiples of MIC.

**TABLE 2:** Average kill metrics on exposure to 10x MIC Cefotaxime stratified on the presence of acquired β-lactamases^*a*^

	Duration of bacteriostasis	Half-life in first kill phase	MDK_99_	Proportion of cells in second kill phase
	(T_0_ – min)	(ln2/k_1_ – min)	(min)	(*P* – n/10^5^ CFU)
β-lactamase neg.	53.1 (44.0-62.2)	4.1 (2.0-6.3)	70.7 (52.2-89.2)	7.7 (2.1-13.3)
TEM-1	62.8 (33.8-91.9)	3.5 (0.6-6.4)	80.6 (64.5-96.7)	9.0 (0.5-17.5)
OXA-1	57.8 (36.6-79.1)	3.8 (0.9-6.7)	84.2 (69.3-99.1)	34.3 (15.0-53.7)
p-value	NS	NS	NS	0.011

^*a*^ Mean values from 15 clinical isolates; 95% CI is indicated within parentheses. *P*-values were calculated using the Kruskal-Wallis test.

### Killing by fluoroquinolones differs from β-lactams

Two isolates, both producing OXA-1, were highly resistant to ciprofloxacin (Table S3). Therefore, time-kill assays with ciprofloxacin were only done using the remaining 13 clinical isolates and *E. coli* ATCC25922.

In contrast to β-lactam-mediated killing, CIP exposure resulted in an immediate reduction of CFUs without the bacteriostatic phase observed for β-lactams. Modeling with and without a bacteriostatic phase resulted in almost identical fits to the experimental data. An initial bacteriostatic phase, therefore, seems to be a function relating to β-lactam exposure and not a result of assay conditions used in the study.

Metrics of ciprofloxacin kill are compared with the metrics of all β-lactams as a group in Table 3. The half-lives of CFUs in the first kill phase during CIP exposure were comparable with those of the β-lactams, with a mean of 3.2 min obtained for CIP compared with 3.1 min obtained for the β-lactams as a group. The average MDK_99_ for CIP was significantly shorter than for β-lactams, reflecting the absence of a bacteriostatic phase for CIP. The overall faster killing obtained with CIP compared to β-lactams is a result of CIP providing immediate killing in contrast to β-lactams.

**TABLE 3:** Kill metrics of ciprofloxacin compared with β-lactams^*a*^

	Duration of bacteriostasis	Half-life in first kill phase	MDK_99_	Proportion of cells in second kill phase
	(T_0_ – min)	(ln2/k_1_ – min)	(min)	(*P* – n/10^5^ CFU)
CIP	0^*b*^	3.1 (2.2-4.0)	19.3 (14.8-23.7)	7.7 (0.4-14.9)
β-lactams	56.7 (52.2-61.1)	3.2 (2.4-4.0)	75.0 (70.5-79.4)	26.1 (17.6-34.7)
*P*-value	9.8 × 10^−4^	NS	4.9 × 10^−4^	0.012

^*a*^ Mean values from 15 clinical isolates; 95% CI is indicated within parentheses. *P*-values were calculated using the Wilcoxon-signed rank test; *NS* signifies a non-significant result, i.e.*, P* > 0.05.

^*b*^ CIP experimental data was fitted without an initial bacteriostatic phase.

A significantly lower fraction of CFUs underwent killing in the second kill phase during CIP exposure compared with the β-lactams (Table 3). For two of the clinical isolates and for *E. coli* ATCC25922, the proportions of cells undergoing second-phase killing were below the detection limit of the time-kill assay. For the remaining, the average proportions were 7.7/100.000 CFU and 26/100.000 CFU for CIP and β-lactams, respectively. Notably, the isolate that displayed a high persister phenotype to β-lactam-mediated killing only had a small proportion of cells undergoing second-phase killing by CIP.

## DISCUSSION

In this study, we designed a high-resolution time-kill assay and a mathematical model for analysis of kill curves. We used these to provide quantitative measurements of tolerance and persistence for 15 clinical *E. coli* isolates and *E. coli* ATCC25922 when challenged with three different beta-lactam antibiotics and CIP. We found the kill curve data produced to be reproducible and obtained coefficients of variation of <0.30 for all parameters in our mathematical model when performing three biological repetitions of the assay. The coefficients of variation were higher when fitting data with CIP. This is not entirely unexpected, as we designed our assay using beta-lactam antibiotics. During the challenge with CIP, CFU counts in the second kill phase were close to the detection limit of the assay, resulting in fewer accurate time points for modeling the data of this phase of the kill curve.

The reproducibility of the results obtained in the time-kill assay allowed us to investigate the variation in kill metrics among *E. coli* isolates. Excluding outliers, we could establish average kill metrics and associated 95% CI, providing tentative wild-type distributions for each metric (Table 1). Generally, kill metrics were correlated between TZP and CTX, both antibiotics preferentially binding PBP3, but not to MEM, an antibiotic preferentially binding PBP2 (16). Meropenem also showed a significantly shorter duration of the bacteriostatic phase and consequently a shorter MDK_99_ compared with both TZP and CTX, suggesting that differences in antibiotic action impact observed kill metrics. The proportion of cells undergoing second-phase killing was also relatively higher for meropenem compared to the other β-lactams.

Using high-density sampling, we showed that killing by β-lactams was preceded by a bacteriostatic phase. This could not be attributed to quiescence of cells in lag phase, as (i) mock-treated cultures showed exponential growth with expected doubling times (17), and (ii) ciprofloxacin killing in the same assay was modeled in the absence of a lag phase. Instead, we suggest that the bacteriostatic phase is inherent to the action of β-lactams, although it is only rarely reported in the literature (18). Its underreporting may reflect that most time kill assays have been done using only sparse sampling (19–21).

β-lactams induce the SOS response through binding to PBP3, encoded by *fts*I, and subsequent signaling through the DpiBA two-component system to a *rec*A and *lex*A-dependent induction of *sul*A (22). The *sul*A gene product inhibits FtsZ polymerization and inhibits cell division (23), providing temporary relief from β-lactam-mediated cell killing. Indeed, in *E. coli,* kill curves using ampicillin showed a bacteriostatic phase was observed, which was absent when using isogenic strains containing inactivating mutations in either *rec*A, *dpi*A, or *sul*A (22).

We did not observe a bacteriostatic phase with ciprofloxacin, although quinolones also induce the SOS response and cause cell filamentation at concentrations near the MIC (24). We conducted the time-kill assay using ciprofloxacin at 10×, the MIC as determined in a macrodilution assay. At low concentrations, compared with the MIC, the quinolone effect is largely reversible, and the effect is bacteriostatic. At high concentrations as used here, quinolones exert their effect through chromosome fragmentation and induction of reactive oxygen species, resulting in a rapid kill as seen here (25, 26).

For β-lactam-mediated killing, the MDK_99_ is a composite measure determined by both the duration of the bacteriostatic phase and the kill rate in the first kill phase. Because of its composite nature, early high-density sampling is necessary to accurately measure MDK_99_. Sparse sampling in time-kill assays may provide misleading estimates for kill rates if a bacteriostatic phase is not recognized, and thus misidentifies tolerance.

The minimum duration of kill to provide a 3 or 4 log decrease in CFU count (MDK_99.9_ or MDK_99.99_) was proposed as a measure for a persister phenotype (12). With the conditions used here in the time-kill assay, we observed persister frequencies in the range from 10 to 100/100.000 CFU for β-lactams (corresponding to log −3 to log −4) (Table 1). Both MDK_99.9_ and MDK_99.99_ therefore lack specificity and overcall a persister phenotype in our data set.

Using the distribution observed to identify outlier values in kill metrics, only four outliers were identified in 58 combinations. Three isolates showed more rapid killing than the majority. The remaining isolate had a 5×–10× higher proportion of cells undergoing second phase killing compared with the wild-type distribution, indicating a persister phenotype. Overall, this may indicate that clinical isolates with increased antibiotic tolerance or persistence are relatively infrequent. This observation is in line with a previous study investigating the prevalence of tolerance and persistence among *E. coli* isolates (11). This study used the TD test to screen for tolerance and/or persistence against ertapenem, ceftriaxone, and ampicillin. The TD test is a convenient screen test but is unable to discern between tolerance and persistence (10) and may be limited in both sensitivity and specificity (11). The authors found a positive test indicating a persister phenotype in 8.5% of first-time *E. coli* isolates from bloodstream isolates and in 28.6% of *E. coli* isolates from recurrent bloodstream infections. Their findings, in line with our data, indicate that tolerance and/or persistence in unselected strain collections is relatively rare.

The present study was designed to investigate whether tolerance to β-lactam antibiotics was affected by the presence of TEM-1 or OXA-1 β-lactamases. For that purpose, only a limited number of antibiotic classes were included, and our results show that the conclusions made are likely specific to antibiotic classes. Using high concentrations of antibiotics, we were able to discern between relative resistance and tolerance. Under these conditions, we did not observe isolates with increased tolerance. However, some isolates displayed large increases in MICs as measured in the modified broth macrodilution assay compared with the standard broth microdilution assay, indicative of heteroresistance (EC11 and EC15 in Table S3). These were consequently exposed to concentrations of antibiotics in the time-kill assay that are not realistic in human dosing. Furthermore, in the time-kill assay, inoculum cells were maintained in the exponential phase prior to exposure to antibiotics. Thus, the assay is optimized to measure the fraction of spontaneous persister cells, that is, the fraction of persister cells in the absence of environmental triggers (12). In natural infections, such environmental triggers of persistence are present, and such triggered persister cells are likely more frequent than spontaneous persisters (27). Caution is therefore advised if results from *in vitro* experiments such as ours are applied in a clinical context. On the other hand, *in vitro* experiments aimed to identify spontaneous persister cells and uninduced tolerance may be useful for understanding how these phenotypes contribute to antibiotic failure, the development of resistance, and the identification of genetic factors underlying this phenotype. To study the role of antibiotic tolerance and persistence in selected cases of antibiotic treatment failure requires the knowledge of wild-type distributions of kill metrics to interpret data. Our study provides a reference methodology and wild-type distributions of kill metrics that may facilitate investigations of the clinical impact of antibiotic tolerance and persistence.

## MATERIALS AND METHODS

### Clinical isolates

We selected 15 isolates of *E. coli* from a collection of clinical isolates at the Department of Clinical Microbiology, Rigshospitalet, Copenhagen, Denmark, to include five isolates each of TEM-1 β-lactamase producers, OXA-1 β-lactamase producers, and without acquired β-lactamases. All isolates were isolated at the hospital as part of the clinical routine. A full genetic sequence was available for all isolates. Clinical susceptibility to TZP, CTX, cefoxitin, and cefpodoxime was an additional selection criterion. Isolates were excluded if they produced any acquired β-lactamases other than TEM-1 and OXA-1 based on the available sequence data.

### MIC determination

We determined MICs for TZP, CTX, MEM, and CIP of all isolates using microbroth dilution methodology according to the EUCAST guidelines (28, 29). In order to account for the inoculum effect in our time-kill assays (30), we also determined the MIC of each isolate in a modified macrobroth dilution assay using an increased inoculum of 5 × 10^6^ CFU/mL in 1 mL Mueller-Hinton broth (Sigma Aldrich) (29). The macrobroth MIC was read as the lowest concentration of antibiotics without visible growth after 18 h of incubation at 35°C with shaking at 225 rpm (29). The results of both assays can be found in Table S3.

### Time-kill assay

Test strains were grown overnight in Mueller-Hinton broth. They were then diluted 1,000-fold in fresh Mueller-Hinton broth and incubated at 37°C for 3 h to ensure the bacteria had reached the exponential phase. Bacterial cultures were then adjusted to 5 × 10^6^ CFU/mL in 9 mL Mueller-Hinton broth containing the study antibiotic at a concentration of 10× MIC as determined in the modified macrobroth dilution assay. The assay cultures were incubated at 35°C with shaking at 225 rpm.

Bacterial survival was determined as CFU counts at 0, 15, 30, 45, 60, 75, 90, 120, 180, 240, 360, and 480 min. During the first 75 min, antibiotic carry-over was eliminated by dilution as CFU counts were determined by spotting 10-fold dilution series of the sample in 10 µL spots in three replicates onto Mueller-Hinton agar plates. After the first 75 min, antibiotic carry-over was eliminated by washing the samples twice in saline. CFU counts were determined both by spotting the washed sample as above and by inoculating 1 mL of the washed sample into a separate Mueller-Hinton plate to lower the detection limit of the assay. The minimal accurately countable number of CFUs was 30 CFU/mL for samples obtained from 90 min of incubation to the termination of the assay. CFU counts were read after 18 h incubation at 37°C. All time-kill assays were carried out in three biological replicates.

Three different phases were apparent in a typical time-kill assay: First, a bacteriostatic phase without any reduction of CFU counts; second, a phase of rapid reduction in CFU counts; and finally, a phase with a slow reduction in CFU counts. The three phases were modeled mathematically as two populations undergoing exponential killing after a bacteriostatic phase using the following equation:


$$N\left(t\right)=N_{0}x({pxe}^{-k1x\left(t-T0\right)}+\left(1-p\right)xe^{-k2x\left(t-T0\right)})$$

where *N*_0_ is the size of the inoculum, *T*_0_ is the duration of the bacteriostatic phase, *k*_1_ is the kill rate of the population undergoing rapid killing, *k*_2_ is the kill rate of the population undergoing slow killing, and *P* is the proportion of cells undergoing rapid killing (cf. Fig. 1). The model was fitted to experimental data using the Solver function in Excel minimizing the sum of squares of the residuals between observed and calculated log CFU/mL of the samples optimizing the parameters above. The accuracy of the estimates, given by the average coefficient of variation for each isolate across the different parameters, was: 0.16 for *T*_0_, 0.27 for *k*_1_, 0.28 for *k*_2_ and <0.01 for *P* when fitting data for β-lactams and 0.38 for *k*_1_, 0.73 for *k*_2_, and <0.01 for *P* when fitting data for CIP. The proportion of cells undergoing slow, second phase killing was used to estimate persistence. Two estimates for tolerance were used: (i) the half-life of cells undergoing rapid, first-phase killing, and (ii) the minimal duration of kill reducing viable cells by 2 log (MDK_99_). MDK_99_ was calculated from the fitted parameter estimates obtained.

### Statistics

Calculations of means, 95% confidence intervals, and statistical tests were, if not otherwise stated, performed in GraphPad (Prism 10.4.1). Parameters obtained from the mathematical model during beta-lactam exposure were compared using the Friedman test. Isolates deviating more than 2 SD from the mean were excluded from the analysis of the parameter. The Friedman test was followed by post-hoc Wilcoxon signed-rank tests using the Bonferroni correction performed in R (version 4.2.2). Linear regression was used to investigate any correlation between TZP and the other three antibiotics, in addition to any correlation between the initial bacteriostatic phase and the half-life of CFU counts in the first kill phase. The effect of the isolate’s beta-lactamase production was evaluated using the Kruskal-Wallis test with exclusion of all outliers. Wilcoxon signed-rank test comparing CIP and the beta-lactams was performed in R. Outliers and isolates without data for ciprofloxacin were excluded.

## SUPPLEMENTAL MATERIAL

Figures S1 and S2, and Tables S1 to S3 spectrum.01124-25-s0001.docx Figure S1: Growth curve of untreated control in high-resolution time-kill assay. Figure S2: Kill metrics not correlating between antibiotics. Table S1: Average kill metrics on exposure to 10x MIC TZP stratified on the presence of acquired β-lactamases. Table S2: Average kill metrics on exposure to 10x MIC MEM stratified on the presence of acquired β-lactamases. Table S3: Microbroth dilution MICs and modified macrobroth dilution MICs of study isolates.

ASM does not own the copyrights to Supplemental Material that may be linked to, or accessed through, an article. The authors have granted ASM a non-exclusive, world-wide license to publish the Supplemental Material files. Please contact the corresponding author directly for reuse.
