# Supplementary material for: Antibiotic tolerance and persistence in clinical isolates of Escherichia coli evaluated by high-resolution time-kill assays
Source: Microbiol Spectr. 2025 Aug 7;13(9):e01124-25. doi: 10.1128/spectrum.01124-25 (PMC12403604; doi:10.1128/spectrum.01124-25)
Supplement: Figures S1 and S2, and Tables S1 to S3 — Figure S1: Growth curve of untreated control in high-resolution time-kill assay. Figure S2: Kill metrics not correlating between antibiotics. Table S1: Average kill metrics on exposure to 10x MIC TZP stratified on the presence of acquired β-lactamases. Table S2: Average kill metrics on exposure to 10x MIC MEM stratified on the presence of acquired β-lactamases. Table S3: Microbroth dilution MICs and modified macrobroth dilution MICs of study isolates. [file spectrum.01124-25-s0001.docx]

**SUPPLEMENTAL MATERIAL**

**Figure S1: Growth curve of untreated control in high-resolution time-kill assay.**

**
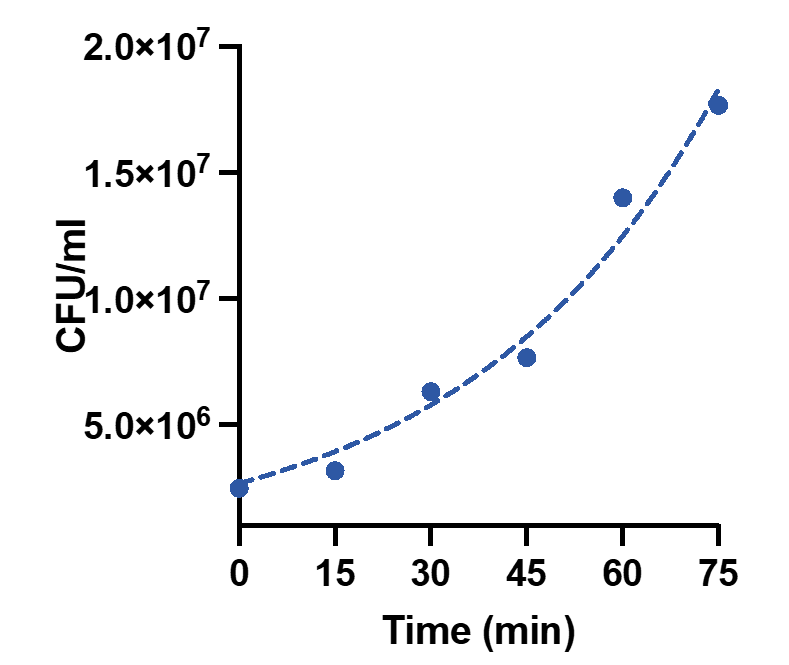
**

**Figure S1:** Exponential growth of a clinical isolate of *E. coli* in the time-kill assay in the absence of antibiotic. Shown are CFU-counts in samples taken during the first 75 minutes of the assay.

**Figure S2: Kill metrics not correlating between antibiotics**


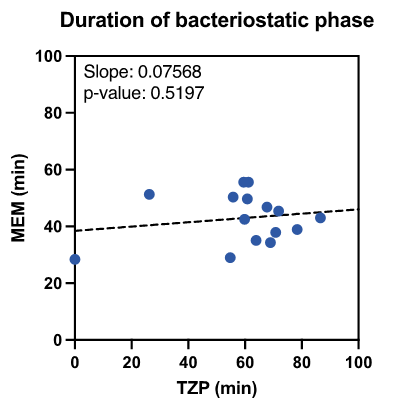

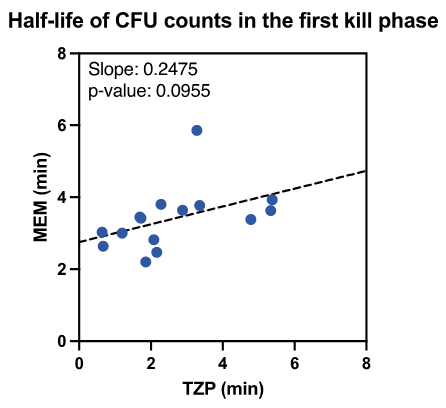

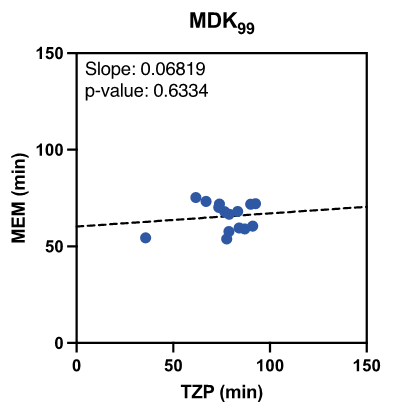

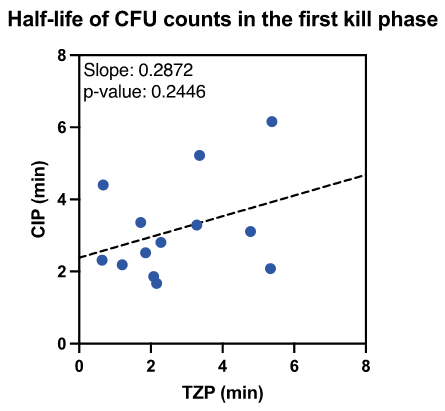

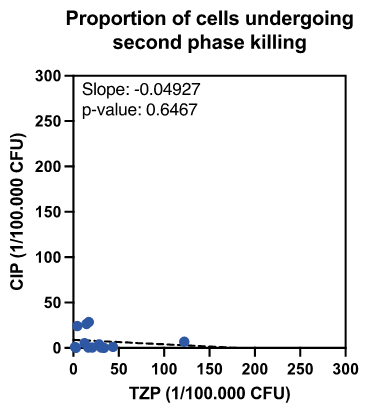

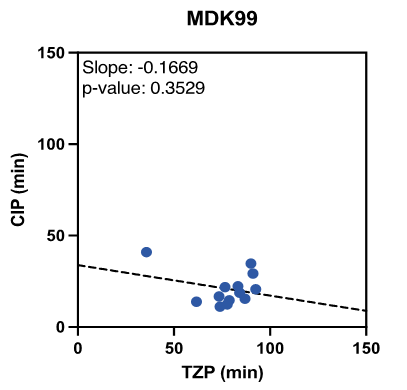


**Figure S2:** XY-plots of kill metrics between TZP and MEM (upper panel) and TZP and CIP (lower panel). No significant association were identified between TZP and MEM with regard to duration of bacteriostasis (*t*_0_), half-life of cells during first kill phase (ln2/*k*_1_), or MDK_99_, or between TZP and CIP with regard to proportion of cells undergoing second phase killing (*p*), half-life of cells during first kill phase (ln2/*k*_1_), or MDK_99_.

**Table S1: Average kill metrics on exposure to 10x MIC TZP stratified on the presence of acquired β-lactamases.**

Duration of Half-life in first MDK_99_ Proportion of cells

bacteriostasis kill phase in second kill phase

(*T*_0_ – min) (ln2/*k*_1_ – min) (min) (*p* – n/10^5^ CFU)

β-lactamase neg. 67.6 (45.7-89.4) 2.8 (0-6.0) 78.6 (63.3-93.8) 21.2 (1.2-41.1)

TEM-1 68.9 (63.2-74.5) 2.5 (0.5-4.6) 81.0 (75.8-86.1) 10.4 (0-24.5)

OXA-1 64.6 (50.0-79.3) 2.6 (0-5.3) 83.1 (69.9-96.3) 28.0 (15.8-40.1)

p-value *NS* *NS* *NS* *NS*

Mean values from 15 clinical isolates; 95% CI is indicated within parenthesis.

p-values were calculated using the Kruskal-Wallis test.

**Table S2: Average kill metrics on exposure to 10x MIC MEM stratified on the presence of acquired β-lactamases.**

Duration of Half-life in first MDK_99_ Proportion of cells

bacteriostasis kill phase in second kill phase

(*T*_0_ – min) (ln2/*k*_1_ – min) (min) (*p* – n/10^5^ CFU)

β-lactamase neg. 43.6 (26.0-61.2) 3.1 (1.8-4.3) 67.6 (59.0-76.2) 59.6 (26.8-92.3)

TEM-1 38.2 (30.1-46.2) 3.4 (2.8-4.0) 59.8 (50.3-69.4) 30.4 (13.7-47.2)

OXA-1 46.7 (34.9-58.5) 3.1 (2.3-3.9) 67.5 (57.7-77.3) 42.9 (0-106)

p-value *NS* *NS* *NS* *NS*

Mean values from 15 clinical isolates; 95% CI is indicated within parenthesis.

p-values were calculated using the Kruskal-Wallis test.

**Table S3: Microbroth dilution MICs and modified macrobroth dilution MICs of study isolates.**

MIC microbroth (mg/L) MIC mod. macrobroth (mg/L)

MLST TZP CTX MEM CIP TZP CTX MEM CIP

*bla*-negative isolates:

Ec01 SLV-295** 4 0.063 0.008 0.016 8 0.5 0.008 0.031

Ec02 404 2 0.063 0.008 0.008 8 0.5 0.008 0.031

Ec03 12 2 0.125 0.031 0.031 4 0.5 0.063 0.031

Ec04 2604 2 0.063 0.031 0.016 4 0.25 0.031 0.008

Ec05 80 2 0.063 0.031 0.016 4 0.5 0.063 0.016

TEM-1-positive isolates:

Ec06 69 2 0.063 0.031 0.016 8 0.25 0.031 0.031

Ec07 141 2 0.063 0.031 0.031 8 0.5 0.031 0.031

Ec08 131 4 0.125 0.008 0.063 32 0.5 0.031 0.031

Ec09 131 2 0.063 0.008 0.25 16 0.125 0.008 0.5

Ec10 196 8 0.063 0.008 0.008 64 0.25 0.031 0.016

OXA-1-positive isolates:

Ec11 131 4 0.25 0.125 >64 64 1 0.125 >64

Ec12 131 4 0.063 0.031 0.031 8 0.25 0.031 0.125

Ec13 10 8 0.125 0.016 0.016 16 0.25 0.031 0.016

Ec14 59 8 0.25 0.016 0.016 16 0.5 0.016 0.016

Ec15* 90 16 0.25 0.008 1 512 4 0.031 >64

*Isolate contained *bla*_TEM-1_ in addition to *bla*_OXA-1_.

**Isolate was a single locus sequence variant of ST-295.
